# Supplementary material for: Insect Collections as an Untapped Source of Bioactive Compounds—Fireflies (Coleoptera: Lampyridae) and Cardiotonic Steroids as a Proof of Concept
Source: Insects. 2021 Jul 31;12(8):689. doi: 10.3390/insects12080689 (PMC8396437; doi:10.3390/insects12080689)
Supplement: Supplementary file 1 [file insects-12-00689-s001.zip › insects-1307065-supplementary.pdf]

insects

MDPI

Insect Collections as an Untapped Source of Bioactive Compounds—Fireflies (Coleoptera: Lampyridae) and Cardiotonic Steroids as a Proof of Concept

Andreas Berger [1,†], Georg Petschenka [2,†,*], Thomas Degenkolb [1], Michael Geisthardt [3] and Andreas Vilcinskas [1,4,*]

Supplementary Materials

Table 1. Beetle specimens analyzed in this study. Months are indicated by Roman numerals. The country of origin was translated into English for ease of interpretation, all other information is presented as on the original label. AB: A. Berger, AE: A. Elbert, DE: D. Erber, MG: M. Geisthardt, WH: W. Höhner, WL: W. Lucht, HV: H. Vogt; coll. AB = collection Andreas Berger, coll. ME = collection Manfred Egger, MfN = Museum für Naturkunde Berlin, SMF = Senckenberg Museum Frankfurt**.**

Sample ID	Family	Species	Subfamily	Collection Date	Location	Collector	Determined	Collection	Sex
60	Lampyridae	*Lamprohiza delarouzei* (Jacquelin du Val, 1859)	Lamprohizinae	n.d.	France, Marseille	Anney	MG	MfN	
52	Lampyridae	*Lamprohiza mulsantii* (Kiesenwetter, 1850)	Lamprohizinae	n.d.	Italy, Imperia Sanremo	Schneider	MG	MfN	♂
53	Lampyridae	*Lamprohiza mulsantii* (Kiesenwetter, 1850)	Lamprohizinae	n.d.	Italy, Imperia Bordighera	Schneider	MG	MfN	♀
64	Lampyridae	*Lamprohiza paulinoi* (Olivier, 1884)	Lamprohizinae	n.d.	Spain	Olivier	MG	SMF	♂
27	Lampyridae	*Lamprohiza splendidula* (Linnaeus, 1767)	Lamprohizinae	14.VI.2007	Greece, Peleponnes Kalavrila Vrachny	M. Egger	MG	coll. ME	♂
63	Lampyridae	*Lamprohiza splendidula* (Linnaeus, 1767)	Lamprohizinae	29.VI.2015	Germany, Hessen	A. Berger	AB	coll. AB	♀

					Maintal, Wachenbuchen				
51	Lampyridae	*Lampyris algerica levigata* (Geisthardt, 1983)	Lampyrinae	15.VI.2011	Tunisia, Tabarka Umg. Golfhotel	M. Egger	MG	coll. ME	♂
70	Lampyridae	*Lampyris algerica levigata* (Geisthardt, 1983)	Lampyrinae	15.VI.2011	Tunisia, Tabarka Umg. Golfhotel	M. Egger	MG	coll. ME	♀
65	Lampyridae	*Lampyris ambigena* (Jacquelin du Val, 1860)	Lampyrinae	n.d.	Italy, Sizilien	Failla	MG	SMF	♂
35	Lampyridae	*Lampyris ambigena* (Jacquelin du Val, 1860)	Lampyrinae	n.d.	Italy, Sizilien Madonio	Geo. C. Kr	MG	MfN	♂
66	Lampyridae	*Lampyris ambigena* (Jacquelin du Val, 1860)	Lampyrinae	n.d.	Italy, Sizilien Madonio	Geo. C. Kr	MG	MfN	♂
67	Lampyridae	*Lampyris angustula* (Fairmaire, 1895)	Lampyrinae	n.d.	Turkey, ca. 60 km SE Tunceli	n.d.	MG	SMF	♂
68	Lampyridae	*Lampyris brutia* (Costa, 1882)	Lampyrinae	1905	Italy, Sant Eufemia d'Aspromonte	Paoanetti	MG	SMF	♂
69	Lampyridae	*Lampyris germariensis* (Jacquelin du Val, 1860)	Lampyrinae	15.IV.1964	Croatia, Spalato	Baganetti	MG	SMF	♂
50	Lampyridae	*Lampyris germariensis* (Jacquelin du Val, 1860)	Lampyrinae	27.VI.1989	Croatia, Istrien Porec	Popp	MG	coll. ME	♂

29	Lampyridae	*Lampyris hellenica* (Geisthardt, 1983)	Lampyrinae	14.VI.1996	Greece, Südpeleponnes Mani, Stoupa	M. Egger	MG	coll. ME	♂
48	Lampyridae	*Lampyris hellenica* (Geisthardt, 1983)	Lampyrinae	10.VI.1996	Greece, Südpeleponnes Mani, Pirgos	M. Egger	MG	coll. ME	♂
71	Lampyridae	*Lampyris lareynii* (Jacquelin du Val, 1859)	Lampyrinae	n.d.	France, Corsica	Lasegn.	MG	SMF	♂
56	Lampyridae	*Lampyris lareynii* (Jacquelin du Val, 1859)	Lampyrinae	n.d.	France, Corsica	n.d.	MG	MfN	♂
22	Lampyridae	*Lampyris noctiluca* (Linnaeus, 1767)	Lampyrinae	08.VII.1989	Austria, Wien Lainzer Tiergarten	M. Egger	MG	coll. ME	♂
23	Lampyridae	*Lampyris noctiluca* (Linnaeus, 1767)	Lampyrinae	30.VI.1989	Italy, Prov. Bozen Auer Castelfeder	M. Egger	MG	coll. ME	♂
24	Lampyridae	*Lampyris noctiluca* (Linnaeus, 1767)	Lampyrinae	30.VII.2004	Bulgaria, Burgas südl. Duni	M. Egger	MG	coll. ME	♂
62	Lampyridae	*Lampyris noctiluca* (Linnaeus, 1767)	Lampyrinae	07.VII.2006	Germany, Sachsen Johnsdorf bei Königswartha	A. Berger	AB	coll. AB	♀
72	Lampyridae	*Lampyris pallida* (Geisthardt, 1987)	Lampyrinae	1920	Malta, Citadelle Pleimes	n.d.	MG	SMF	♂

73	Lampyridae	*Lampyris pseudozenkeri* (Geisthardt, 1999)	Lampyrinae	1899	Turkey, Anatolien Konia	Korb	MG	SMF	♂
74	Lampyridae	*Lampyris pseudozenkeri* (Geisthardt, 1999)	Lampyrinae	1899	Turkey, Anatolien Konia	Korb	MG	SMF	♂
75	Lampyridae	*Lampyris raymondi* (Mulsant & Rey, 1859)	Lampyrinae	n.d.	France, Gallia	unbekannt	MG	SMF	♂
55	Lampyridae	*Lampyris raymondi* (Mulsant & Rey, 1859)	Lampyrinae	01.VI.1944	France, Umg. Toulon	F. Ermisch	MG	MfN	♂
76	Lampyridae	*Lampyris raymondi* (Mulsant & Rey, 1859)	Lampyrinae	01.VI.1944	France, Umg. Toulon	F. Ermisch	MG	MfN	♂
77	Lampyridae	*Lampyris sardiniae* (Geisthardt, 1987)	Lampyrinae	30.V.1995	Italy, Sardinien Capo Coda, Cavallo	M. Egger	MG	coll. ME	♂
30	Lampyridae	*Lampyris sardiniae* (Geisthardt, 1987)	Lampyrinae	19.V.1995	Italy, Sardinien Capo Coda	M. Egger	MG	coll. ME	♂
31	Lampyridae	*Lampyris sardiniae* (Geisthardt, 1987)	Lampyrinae	10-24.VI.2005	Italy, Sardinien Porto Pollo	R. Lubi	MG	coll. ME	♂
32	Lampyridae	*Lampyris sardiniae* (Geisthardt, 1987)	Lampyrinae	01.V.1914	Italy, Sardinien Oristano	Geisler	MG	MfN	♂
20	Lampyridae	*Lampyris zenkeri* (Germar, 1817)	Lampyrinae	19.VII.2006	Greece, Chalkidiki n. Silhonia Plana	M. Egger	MG	coll. ME	♂

21	Lampyridae	*Lampyris zenkeri* (Germar, 1817)	Lampyrinae	23.VII.-05.08.1990	Greece, Prov. Larisa Stonio	M. Egger	MG	coll. ME	♂
78	Lampyridae	*Lampyris zenkeri* (Germar, 1817)	Lampyrinae	11.VII.1998	Greece, Preveza Kanali	M. Egger	MG	coll. ME	♂
28	Lampyridae	*Lampyroidea achaiaca* (Geisthardt, 1999)	Lamprohizinae	13.VI.2007	Greece, Peleponnes Kalavrita, Abzw. Kerpini	M. Egger	MG	coll. ME	♂
49	Lampyridae	*Lampyroidea achaiaca* (Geisthardt, 1999)	Lamprohizinae	13.VI.2007	Greece, Peleponnes Kalavrita, Abzw. Kerpini	M. Egger	MG	coll. ME	♂
54	Lampyridae	*Lampyroidea dispar* (Fairmaire, 1857)	Lamprohizinae	VI.1902	Iran, Ala-Dagh. Bodschnurd 1033m	Hauser	MG	MfN	♂
61	Lampyridae	*Lampyroidea syriaca* (Costa, 1875)	Lamprohizinae	n.d.	Syria, Küste	J. Greiner	MG	MfN	♂
18	Lampyridae	*Luciola (Luciola) italica* (Linnaeus, 1767)	Luciolinae	26.VI.1990	Italy, Prov. Bozen Kalterersee	M. Egger	MG	coll. ME	♂
19	Lampyridae	*Luciola (Luciola) italica* (Linnaeus, 1767)	Luciolinae	22.VII.1989	Italy, Prov. TV Miane Campea	M. Egger	MG	coll. ME	♂
16	Lampyridae	*Luciola (Luciola) lusitanica* (Charpentier, 1825)	Luciolinae	31.VII.2014	Italy, Piemont, Laux Talhang nachts	A. Berger	AB	coll. AB	♂

insects

MDPI

17	Lampyridae	*Luciola (Luciola) lusitanica* (Charpentier, 1825)	Luciolinae	01.VIII.2014	Italy, Piemont, Villar Perosa ca.500m ü NN, Flussufer	A. Berger	AB	coll. AB	♂
57	Lampyridae	*Luciola (Luciola) novaki* (Muller, 1946)	Luciolinae	28.IV.1936	Bar Mont.	Novak	MG	MfN	♂
79	Lampyridae	*Nyctophila bonvouloirii* (Jacquelin du Val, 1860)	Lamyprinae	n.d.	Italy, Sizilien	n.d.	MG	SMF	♂
80	Lampyridae	*Nyctophila heydeni* (Olivier, 1884)	Lamyprinae	n.d.	Spain, Balearen	Wili.	MG	SMF	♂
81	Lampyridae	*Nyctophila libani* (Laporte de Castelnau, 1833)	Lamyprinae	n.d.	Syria	n.d.	MG	SMF	♂
82	Lampyridae	*Nyctophila maculicollis* (Fairmaire, 1866)	Lamyprinae	n.d.	Iran, Masanderan	Reitter	MG	SMF	♂
83	Lampyridae	*Nyctophila molesta* (Jacquelin du Val, 1859)	Lamyprinae	n.d.	Italy, boreal	n.d.	MG	SMF	♂
58	Lampyridae	*Nyctophila molesta* (Jacquelin du Val, 1859)	Lamyprinae	22.VII.1910	Italy, Liguria Borghetto	n.d.	MG	MfN	♂
84	Lampyridae	*Nyctophila reichii* (Jacquelin du Val, 1859)	Lamyprinae	1900	AK Chehir ?	Korb.	MG	SMF	♂

25	Lampyridae	*Nyctophila reichii* (Jacquelin du Val, 1859)	Lamyprinae	18.VI.2010	Spain Prov. Granada Benamaurel	H. Rietz	MG	coll. ME	♂
26	Lampyridae	*Nyctophila reichii* (Jacquelin du Val, 1859)	Lamyprinae	n.d.	n.d.	J. Greiner	MG	MfN	♂
85	Lampyridae	*Pelania mauritanica* (Linnaeus, 1767)	Lamyprinae	n.d.	Algeria, Ain Fezza	n.d.	MG	SMF	♂
33	Lampyridae	*Pelania mauritanica* (Linnaeus, 1767)	Lamyprinae	n.d.	Algeria, Oran Lalla-Maghrnia	V.M. Duchon	MG	MfN	♂
59	Lampyridae	*Pelania mauritanica* (Linnaeus, 1767)	Lamyprinae	n.d.	Algeria, Oran Lalla-Maghrnia	V.M. Duchon	MG	MfN	♂
86	Lampyridae	*Phosphaenus hemipterus* (Goeze, 1777)	Lamyprinae	25.VI.2016	Germany, Witzenhausen NSG Roßbach	A. Berger	AB	coll. AB	♂
34	Lampyridae	*Phosphaenus hemipterus* (Goeze, 1777)	Lamyprinae	27.VI.1932	Austria, Voralberg Feldkirch	Dr. Feige	MG	MfN	♂
87	Lampyridae	*Phosphaenus hemipterus* (Goeze, 1777)	Lamyprinae	27.VI.1932	Austria, Voralberg Feldkirch	Dr. Feige	MG	MfN	♂
36	Lycidae	*Dictyoptera aurora* (Herbst, 1874)	Erotinae	06.IV.1957	Germany, Hessen Groß-Gerau	H. Vogt	HV	SMF	/
42	Lycidae	*Dictyoptera aurora* (Herbst, 1874)	Erotinae	04.VI.2015	Austria, Bad	A. Berger	WH	coll. AB	/

					Eisenkappel Hochobir auf Blüte				
38	Lycidae	*Erotides cosnardi* (Chevrolat, 1839)	Erotinae	11.VI.1958	Germany, NRW, Siebengebirge Ölberg	W. Lucht	WL	Senckenberg Museum	/
41	Lycidae	*Erotides cosnardi* (Chevrolat, 1839)	Erotinae	11.VI.2007	Germany, MV, Thissow Strand angespült	A. Berger	AB	coll. AB	/
44	Lycidae	*Lopheros rubens* (Gyllenhal, 1817)	Erotinae	23.VII.1963	Austria, Kärnten Zellpfarre	A. Elbert	AE	coll. AB	/
45	Lycidae	*Lopheros rubens* (Gyllenhal, 1817)	Erotinae	27.VII.1962	Austria, Kärnten Zellpfarre	A. Elbert	AE	coll. AB	/
39	Lycidae	*Lygistopterus sanguineus* (Linnaeus, 1758)	Calochrominae	05.VI.2008	Germany, Hessen Wüstensachsen, Stirnberg	A. Berger	AB	coll. AB	/
43	Lycidae	*Lygistopterus sanguineus* (Linnaeus, 1758)	Calochrominae	10.VI.2006	Germany, Sachsen Neschwitz	A. Berger	AB	coll. AB	/
37	Lycidae	*Platycis minutus* (Fabricius, 1787)	Erotinae	06.VII.1981	Germany, Fränk. Schweiz Rabeneck	D. Erber	DE	Senckenberg Museum	/
47	Lycidae	*Platycis minutus* (Fabricius, 1787)	Erotinae	16.IX.1967	Germany, Eifel Kyllburg	W. Lucht	WL	Senckenberg Museum	/

40	Lycidae	*Pyropterus nigroruber* (De Geer, 1774)	Erotinae	11.VII.2015	Germany, Zwingenberg NSG Orbishöhe	A. Berger	AB	coll. AB	/

Table 2. Pseudomolecular ions of lucibufagins from firefly specimens that were positively screened via HPLC-DAD (subset).

Sample	Species	*m/z*	t_R major components	*m/z*	t_R minor components
LK2	*P. pyralis*	449,2175<br>491,2272<br>533,2363	10,3-10,8<br>12,2-12,5<br>13,3-13,7	547,2471<br>561,2634	14,6-14,7<br>15,7-15,8
51	*L. algerica levigata* ♂	449,2188<br>447,2047<br>433,2253<br>491,2278<br>547,2557	10,2-11,2<br>11,8-12,0<br>12,1-12,2<br>11,6-12,0<br>15,4-15,7	/	/
70	*L. algerica levigata* ♀	449,2163<br>433,2234	9,9-10,6<br>10,9-12,4	505,2433<br>561,2660<br>563,2511<br>491,2251	11,5-12,0<br>12,8-13,0<br>12,3-12,7<br>12,3-12,7
35	*L. ambigena* ♂	449,2188<br>433,2242<br>435,2397<br>447,2044	9,8-10,6<br>10,1-10,3<br>10,6-10,7<br>11,8-12,0	491,2260<br>549,2687<br>533,2394	11,0-11,6<br>11,0-11,2<br>13,0-13,3
66	*L. ambigena* ♂	449,2146<br>433,2200<br>447,2000	9,7-10,1<br>10,1-10,3<br>11,4-12,3	491,2214<br>549,2621<br>477,2190<br>533,2771	10,9-11,4<br>10,9-11,4<br>10,9-11,4<br>13,2-13,5
67	*L. angustula* ♂	449,2134<br>565,2611<br>491,2238	9,8-10,7<br>11,3-11,7<br>12,4-12,6	433,2196<br>563,2460<br>549,2269	10,8-11,3<br>12,4-12,6<br>13,2-13,6
68	*L. brutia* ♂	449,2151<br>565,2610<br>433,2212<br>491,2613<br>549,2666<br>533,2668	10,2-10,5<br>10,9-11,3<br>11,3-11,7<br>11,7-12,0<br>11,7-12,4<br>12,9-13,1	477,2426<br>447,2007	10,9-12,0<br>11,7-12,0
69	*L. germariensis* ♂	449,2142<br>491,2238<br>447,2013	9,7-10,1<br>10,9-11,3<br>11,2-11,5	433,2045<br>563,2473<br>477,2177<br>505,2017<br>593,2877<br>533,2377	10,3-10,6<br>10,5-11,0<br>10,9-11,3<br>11,5-11,9<br>11,8-12,4<br>13,1-13,4
29	*L. hellenica* ♂	563,2485	13,1-13,5	433,2404<br>449,2187<br>505,2046<br>447,2049<br>491,2261<br>549,2293	10,2-10,3<br>10,5-10,7<br>11,1-11,5<br>11,5-11,7<br>12,5-12,7<br>12,5-12,7
48	*L. hellenica* ♂	447,2059<br>563,2514<br>491,2273<br>549,2340	10,0-10,2<br>13,2-13,4<br>12,4-12,7<br>12,4-12,7	449,2173<br>505,2070	10,4-10,6<br>11,1-11,5

56	*L. lareynii* ♂	449,2184	9,8-10,7	447,2057	10,4-10,7
71	*L. lareynii* ♂	449,2161<br>447,2034	9,8-10,7<br>11,8-12,2	505,2345<br>477,2429<br>491,2263<br>433,2229<br>533,2401<br>547,2503	11,3-12,0<br>11,3-11,7<br>11,3-11,7<br>11,8-12,2<br>11,8-13,2<br>13,1-13,4
22	*L. noctiluca* ♂	449,2160<br>491,2250<br>433,2234<br>477,2448<br>447,2025<br>529,2073	10,1-11,1<br>10,9-11,1<br>11,4-11,6<br>11,4-11,6<br>11,7-12,0<br>13,4-13,7	505,2103<br>533,2366<br>547,2147	11,1-11,4<br>11,7-12,0<br>13,4-13,7
23	*L. noctiluca* ♂	449,2153<br>433,2225<br>491,2259	9,8-10,5<br>11,4-11,6<br>12,3-12,6	447,2017	11,2-11,4
24	*L. noctiluca* ♂	575,2496<br>593,2568	14,7-15,1<br>14,2-14,4	449,2158<br>491,2258<br>505,2039<br>549,2307	10,4-10,9<br>10,4-10,9<br>10,5-11,1<br>11,8-12,1
62	*L. noctiluca* ♀	449,2186<br>433,2255	9,8-10,4<br>10,7-13,4	491,2280<br>447,2051	12,3-12,8<br>10,7-11,2
72	*L. pallida* ♂	449,2172<br>491,2275	9,7-10,7<br>11,3-12,6	477,2435<br>447,2050<br>433,2236<br>533,2421	10,9-11,3<br>11,3-11,6<br>11,7-12,1<br>12,7-13,2
73	*L. pseudozenkeri* ♂	/	/	449,2053<br>491,2262<br>565,2628<br>433,2243<br>505,2404<br>533,2403<br>563,2506	9,5-11,6<br>10,9-12,6<br>10,9-11,3<br>11,3-11,6<br>11,7-12,0<br>12,3-12,6<br>12,3-12,6
74	*L. pseudozenkeri* ♂	/	/	433,2358<br>449,2145<br>491,2258<br>565,2620<br>533,2394<br>563,2497	10,5-11,7<br>10,5-10,8<br>11,0-11,7<br>11,3-11,7<br>12,4-12,8<br>12,4-12,8
55	*L. raymondi* ♂	449,2182<br>491,2281<br>563,2520	9,9-10,1<br>11,4-12,5<br>11,4-11,6	477,2460<br>533,2453<br>447,2060<br>433,2261	11,8-12,1<br>11,8-12,1<br>11,8-12,1<br>11,8-12,1
75	*L. raymondi* ♂	/	/	449,2152<br>447,2038	10,5-11,0<br>10,7-10,9
76	*L. raymondi* ♂	449,2167<br>565,2632<br>491,2271	9,8-10,8<br>10,8-11,8<br>11,5-12,8	433,2254<br>477,2445<br>549,2670<br>447,2039	11,0-11,5<br>11,5-11,8<br>11,8-12,3<br>11,8-12,3

				563,2509<br>533,2433	12,3-12,8<br>12,8-13,5
30	*L. sardiniae* ♂	449,2164<br>477,2481<br>491,2267<br>533,2356	10,2-10,8<br>11,3-11,6<br>12,3-12,5<br>13,2-13,5	447,2034<br>563,2507	10,6-10,8<br>14,1-14,3
31	*L. sardiniae* ♂	565,2668	11,3-11,5	435,2395<br>449,2205<br>433,2239<br>491,2339<br>477,2478<br>533,2699	10,7-10,9<br>10,7-10,9<br>11,3-11,5<br>12,8-13,0<br>11,5-11,7<br>12,8-13,0
77	*L. sardiniae* ♂	477,2471	11,4-11,7	449,2239<br>491,2278<br>565,2650<br>563,2519<br>547,2512<br>533,2424	9,9-10,2<br>11,4-11,7<br>11,4-11,7<br>14,1-14,3<br>14,1-14,3<br>13,3-13,8
20	*L. zenkeri* ♂	491,2261	11,4-11,6	447,2040<br>449,2161	10,0-10,2<br>10,3-10,5
21	*L. zenkeri* ♂	449,2160<br>491,2264<br>491,2263<br>433,2234<br>447,2035	10,2-11,2<br>11,0-11,2<br>11,4-12,1<br>11,3-11,6<br>11,8-12,1	533,2368<br>477,2447	11,1-11,4<br>11,3-11,6
78	*L. zenkeri* ♂	491,2286<br>449,2186<br>533,2422	11,0-11,2<br>11,2-11,5<br>12,2-12,5	433,2293<br>447,2044<br>477,2450<br>563,2533<br>547,2196<br>593,2941	10,5-10,9<br>10,5-10,9<br>11,5-11,7<br>13,9-14,4<br>13,9-14,4<br>12,5-12,8
80	*N. heydeni* ♂	449,2186	9,8-10,2	447,2046	11,0-11,5
81	*N. libani* ♂	/	/	433,2255<br>477,2474	10,4-10,6<br>10,7-11,0
82	*N. maculicollis* ♂	449,2184<br>447,2066	8,9-10,2<br>10,4-10,8	433,2243<br>549,2705	11,0-11,5<br>11,8-12,3
58	*N. molesta* ♂	449,2188<br>491,2287<br>447,2053<br>593,2975<br>491,2312	9,8-10,6<br>10,9-11,0<br>11,3-11,4<br>11,4-11,6<br>12,3-12,6	477,2446<br>533,2591	11,0-11,2<br>13,3-13,5
83	*N. molesta* ♂	449,2196<br>433,2270<br>447,2064	9,7-11,7<br>11,2-11,7<br>11,8-12,2	491,2292<br>477,2493<br>593,2954	11,0-12,2<br>11,2-11,7<br>11,8-12,2
25	*N. reichii* ♂	449,2178	10,3-10,5	491,2262<br>447,2039<br>433,2240	11,3-12,1<br>11,3-11,6<br>11,7-12,1

insects MDPI

26	*N. reichii* ♂	449,2175<br>447,2030<br>491,2251<br>533,2368	9,8-10,5<br>11,8-12,0<br>11,8-12,0<br>13,2-13,5	565,2646<br>433,2226	11,4-11,5<br>11,4-11,5
84	*N. reichii* ♂	449,2204<br>447,2069	10,2-11,3<br>11,8-12,2	491,2314<br>433,2280<br>565,2671<br>533,2443<br>547,2497	10,9-12,7<br>11,3-11,7<br>11,3-11,7<br>12,3-13,4<br>13,9-14,1
33	*P. mauritanica* ♂	449,2151<br>449,2199<br>449,2100	9,7-10,1<br>10,2-10,6<br>11,8-12,1	447,2052<br>565,2676	11,8-12,1<br>10,7-11,0
59	*P. mauritanica* ♂	449,2193<br>447,2050	10,2-11,2<br>11,4-12,1	433,2251	10,9-11,2
85	*P. mauritanica* ♂	449,2210<br>447,2073<br>491,2310	9,8-10,6<br>11,5-12,3<br>11,5-12,3	433,2272<br>565,2675<br>549,2706<br>563,2564<br>533,2636	11,5-12,3<br>11,5-12,3<br>12,4-12,9<br>12,4-12,9<br>12,4-12,9
34	*P. hemipterus* ♂	/	/	491,2268<br>533,2386<br>433,2235	11,0-11,8<br>13,2-14,6<br>12,0-12,2
86	*P. hemipterus* ♂	/	/	477,2716<br>491,2308<br>565,2674<br>433,2282<br>549,2729<br>533,2452	11,3-11,7<br>11,3-12,2<br>11,3-11,7<br>11,7-12,2<br>11,7-12,2<br>13,2-13,5
87	*P. hemipterus* ♂	/	/	491,2312<br>433,2281<br>549,2719<br>565,2690<br>533,2453	10,9-12,2<br>11,7-12,2<br>11,7-12,2<br>11,7-12,2<br>12,6-13,0

insects MDPI

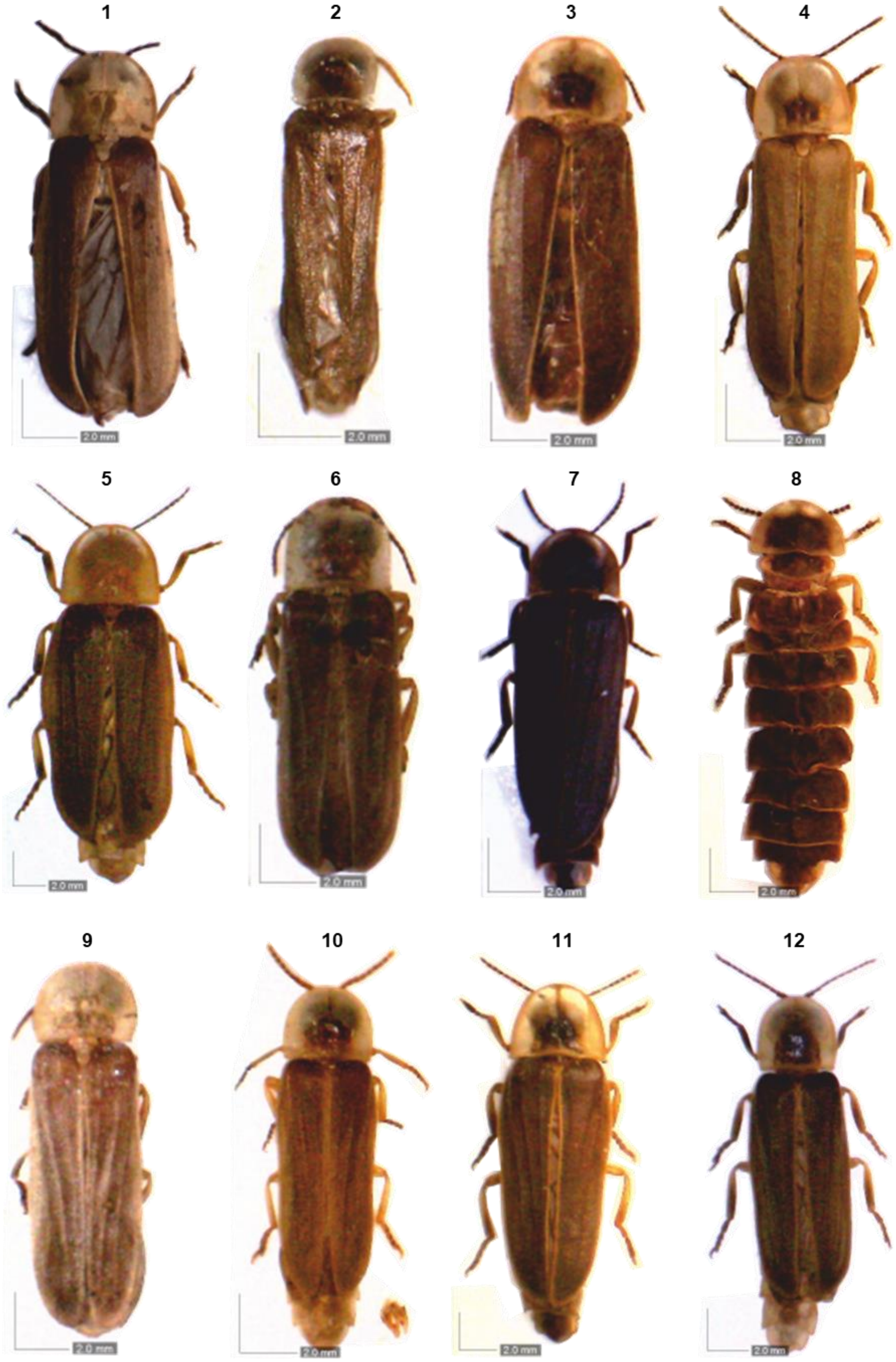

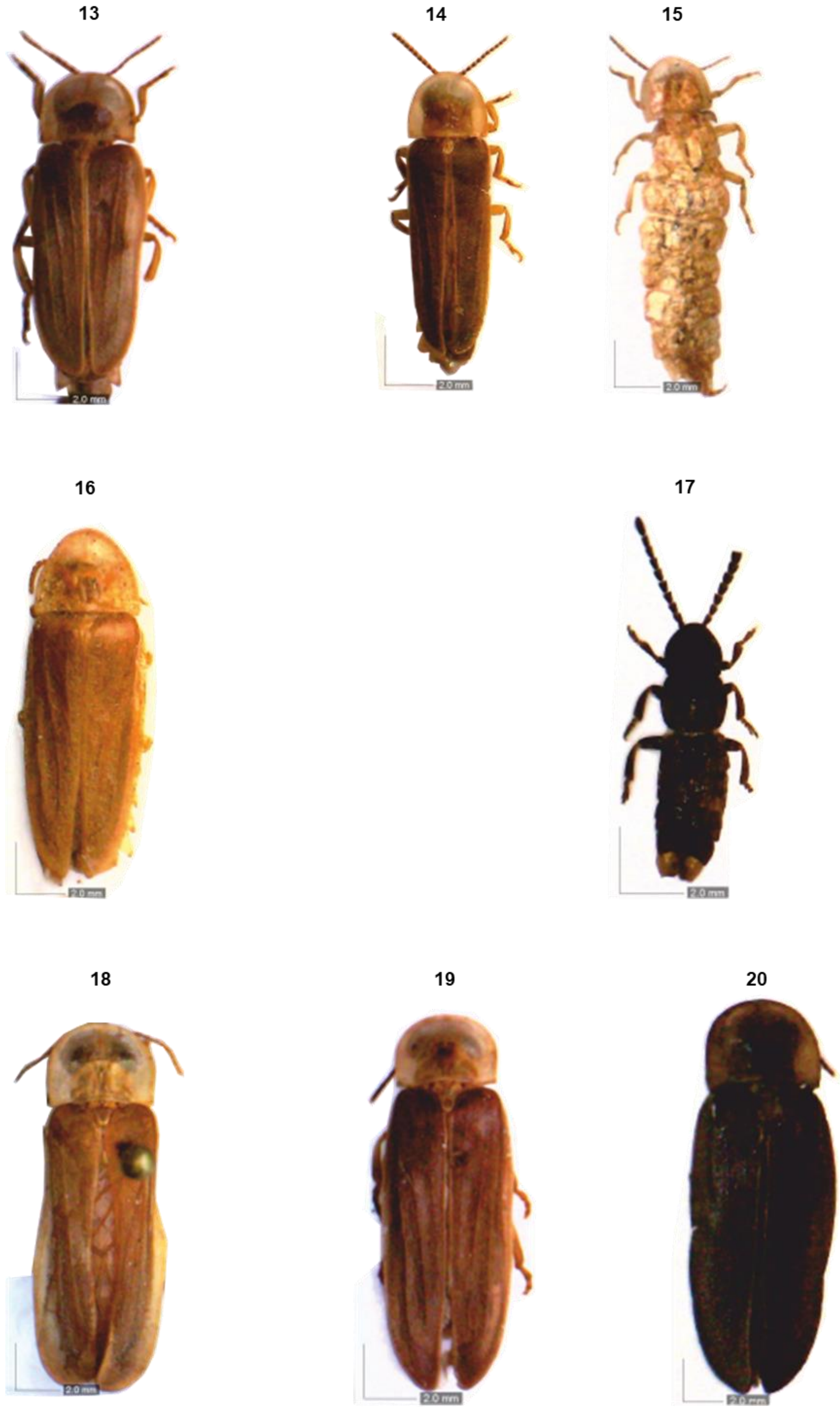
13
14
15
16
17
18
19
20
2.0 mm

insects

21

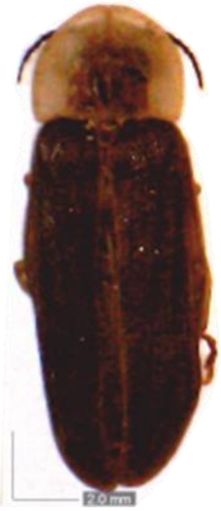

22

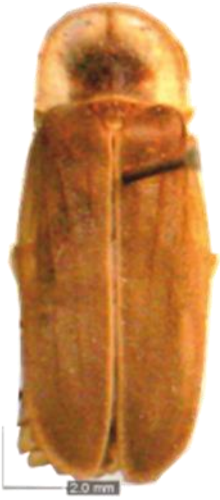

23

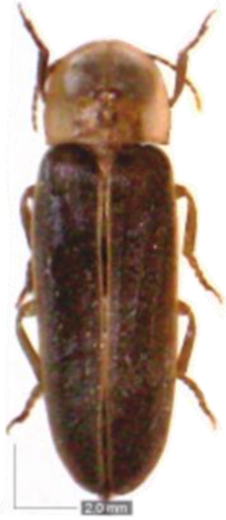

24

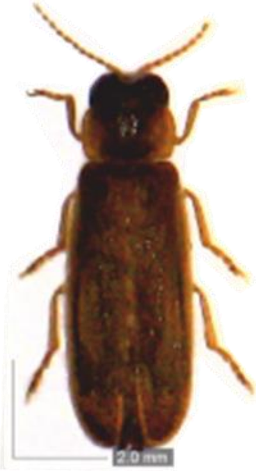

25

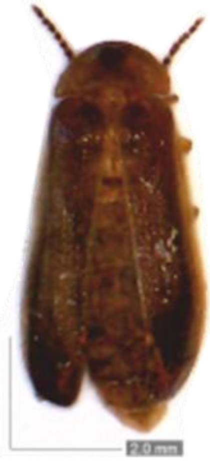

26

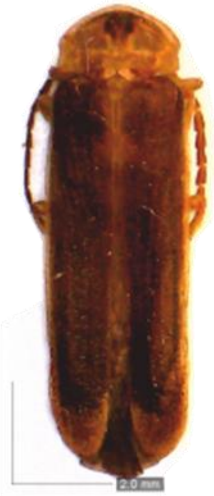

27

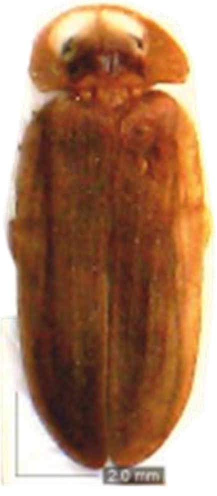

28

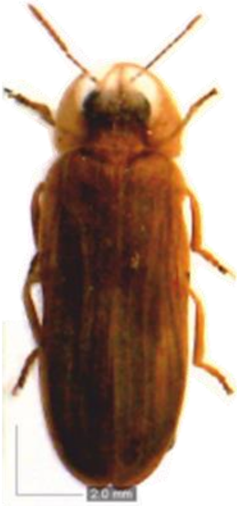

29

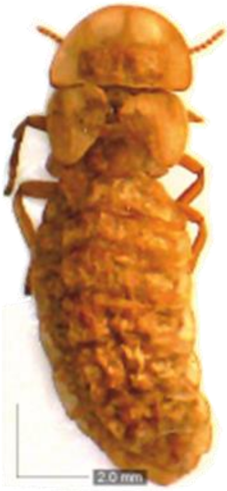

30

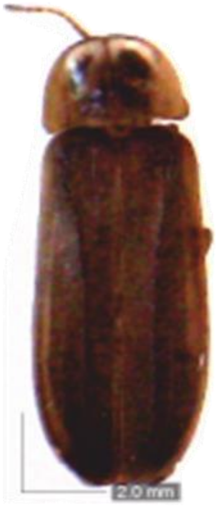

insects

31

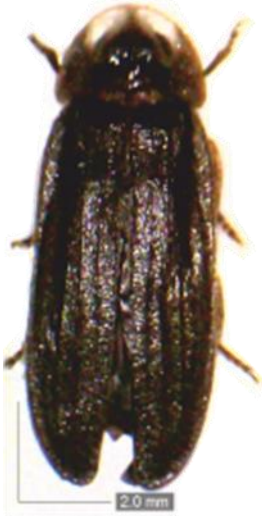

32

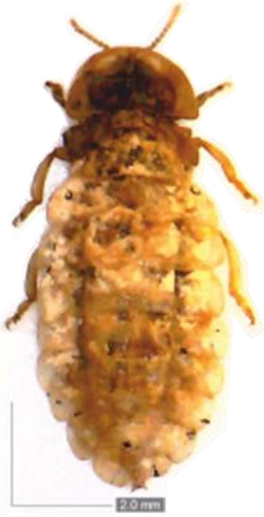

35

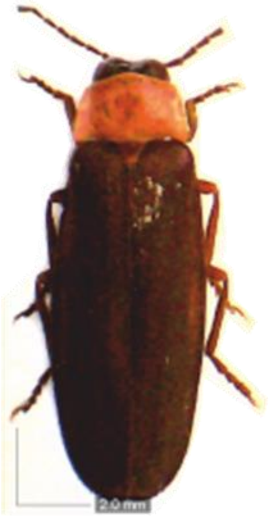

33

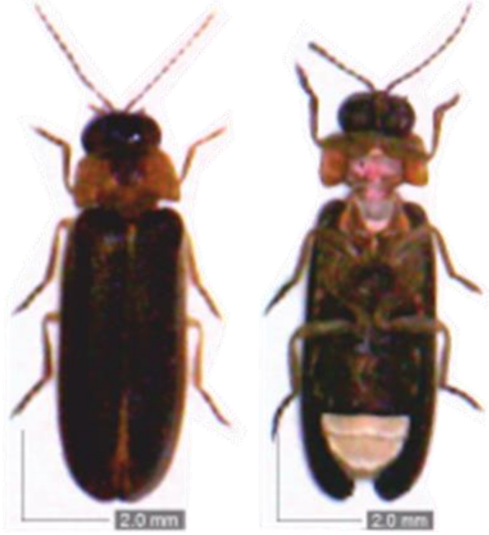

34

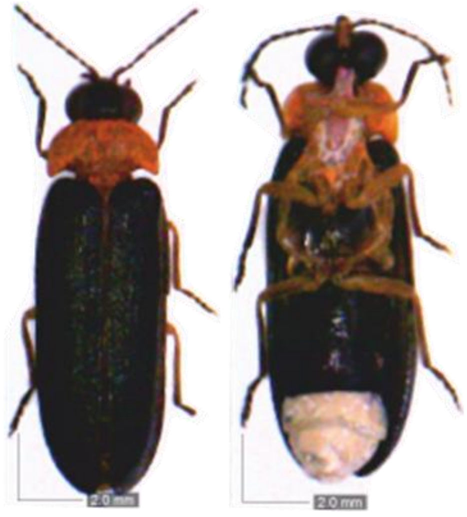

36

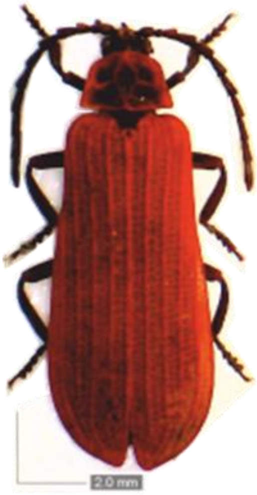

37

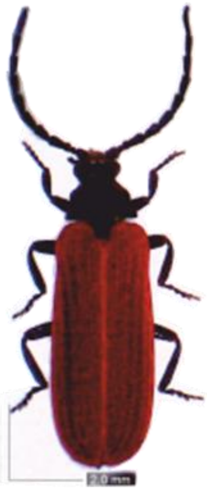

38

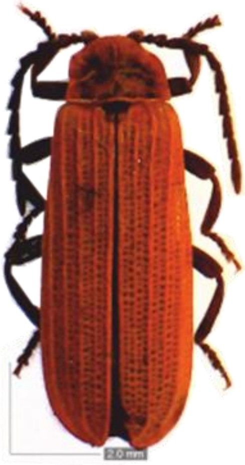

insects MDPI

Figure S1. Specimens after methanol extraction. 1, *Lampyris ambigena* ♂; 2, *L. angustula* ♂; 3, *L. brutia* ♂; 4, *L. germariensis* ♂; 5, *L. hellenica* ♂; 6, *L. lareynii* ♂; 7, *L. noctiluca* ♂; 8, *L. noctiluca* ♀; 9, *L. pallida* (paratype) ♂; 10, *L. pseudozenkeri* ♂; 11, *L. raymondi* ♂; 12, *L. sardiniae* ♂; 13, *L. zenkeri* ♂; 14, *L. algerica levigata* ♂; 15, *L. algerica levigata* ♀; 16, *Pelania mauritanica* ♂; 17, *Phosphaenus hemipterus* ♂; 18 *Nyctophila bonvouloirii* ♂; 19, *N. heydeni* ♂; 20, *N. libani* ♂; 21, *N. maculicollis* ♂; 22, *N. molesta* ♂; 23, *N. reichii* ♂; 24, *Lampyroidea achaiaca* ♂; 25, *L. dispar* ♂; 26, *L. syriaca* ♂; 27, *Lamprohiza delarouzei* ♂; 28, *L. mulsantii* ♂; 29, *L. mulsantii* ♀; 30, *L. paulinoi* ♂; 31, *L. splendidula* ♂; 32 *L. splendidula* ♀; 33, *Luciola italica* ♂; 34, *L. lusitanica* ♂; 35, *L. novaki* ♂; 36, *Dictyoptera aurora*; 37, *Pyropterus nigroruber*; 38, *Lopheros rubens*; 39, *Platycis minutes*; 40, *Erotides cosnardi*; 41, *Lygistopterus sanguineus*.
